# Factors Associated with Prolonged RT-PCR SARS-CoV-2 Positive Testing in Patients with Mild and Moderate Forms of COVID-19: A Retrospective Study

**DOI:** 10.3390/medicina58060707

**Published:** 2022-05-26

**Authors:** Nicoleta Stefania Motoc, Victoria-Maria Ruta, Milena Adina Man, Rodica Ana Ungur, Viorela Mihaela Ciortea, Laszlo Irsay, Andrea Nicola, Dan Valean, Lia Oxana Usatiuc, Ileana Rodica Matei, Ileana Monica Borda

**Affiliations:** 1 Department of Medical Sciences-Pulmonology, Faculty of Medicine, University of Medicine and Pharmacy, 8 Victor Babeș Street, 400012 Cluj-Napoca, Romania; motoc_nicoleta@yahoo.com; 2 “Leon Daniello” Clinical Hospital of Pneumophtysiology, 400371 Cluj-Napoca, Romania; victoria.suteu@yahoo.com; 3 “Iuliu-Hațieganu” PhD Student, Doctoral School, University of Medicine and Pharmacy, 8 Victor Babeș Street, 400012 Cluj-Napoca, Romania; usatiuc.lia.oxana@elearn.umfcluj.ro; 4 Department of Medical Specialties, Faculty of Medicine, ”Iuliu-Hațieganu” University of Medicine and Pharmacy, 8 Victor Babeș Street, 400012 Cluj-Napoca, Romania; ungurmed@yahoo.com (R.A.U.); viorela.ciortea@yahoo.com (V.M.C.); irsaylaszlo@gmail.com (L.I.); monicampop@yahoo.fr (I.M.B.); 5 Department of Molecular Sciences, Faculty of Medicine, University of Medicine and Pharmacy, 8 Victor Babeș Street, 400012 Cluj-Napoca, Romania; andreeanicola.pmr@gmail.com; 6 Department of General Surgery, Fifth Medical Clinic, No 11 Tăbăcarilor Street, 400139 Cluj-Napoca, Romania; valean.d92@gmail.com; 7 Plastic Surgery Department, Faculty of Medicine, “Iuliu-Hațieganu” University of Medicine and Pharmacy, 8 Victor Babeș Street, 400012 Cluj-Napoca, Romania; irmatei@yahoo.com

**Keywords:** COVID-19, prolonged viral load, viral clearance, risk factors

## Abstract

*Background and objectives:* This article aims to evaluate the number of days necessary for patients with mild and moderate forms of COVID-19 to reach undetectable levels of SARS-CoV-2 RNA in the upper respiratory tract specimens. As a secondary objective, we sought to establish a correlation between different conditions associated with longer viral load as this could result in a longer period of contagion and infectivity. *Materials and Methods:* It is a retrospective study. A total of 70 patients with confirmed mild and moderate forms of COVID-19 were enrolled in our study. *Results:* Number of days with traceable viral load was 25.93 (±6.02) days in patients with mild COVID-19 and 26.97 (±8.30) in moderate form (*p* = 0.72). Age, male gender, and obesity, along with several chronic conditions (cardiac, liver, renal, and neurological disease), were associated with prolonged positive RT-PCR test from the nasal swab (therefore prolonged viral load). These are in general, risk factors for severe forms of COVID-19. *Conclusions:* There are several conditions associated with prolonged positive RT-PCR in mild and moderate forms of COVID-19. As to why and what is the significance of it remains to be studied.

## 1. Introduction

In March 2020, the COVID-19 pandemic challenged humankind. In the face of the new and unknown disease, health care systems all over the world reacted promptly to prevent disease dissemination and poor outcomes once the disease has set in. In Romania, at the beginning of the pandemic, hospitalization was compulsory for all confirmed COVID-19 cases, regardless of the severity of the disease [1]. If a patient was tested positive for COVID-19, he had to be hospitalized in order to prevent the spreading of the disease and to benefit from a closer surveillance. The patients could be discharged only if they had two consecutive negative reverse transcription–polymerase chain reaction (RT-PCR) tests at 24 h intervals. This led to prolonged hospitalization due to inconsistent COVID-19 tests results [2]. During the last two years as we got to a better understanding of the disease and its mechanisms, we hospitalized only the severe and critical form of COVID-19, or patients with risks factors for a poor outcome. As of February 2022, the quarantine has been reduced to 5 days for patients that have been vaccinated and 7 days for those that have not received the vaccine [3]. From the existing data, we know that the incubation period for severe acute respiratory syndrome caused by SARS-CoV-2 ranges from 1 to 14 days, with a mean of 5 to 6 days [4]. The viral load persists, in general, up to 8 days in patients with mild and moderate form of disease and up to 24 days in patients with more severe forms of diseases [5,6,7]. The highest viral loads are observed in the beginning of disease onset and a few days after symptoms onset, up to seven days with levels slowly decreasing over the next one to three weeks [8]. The duration of viral RNA shedding is variable, and a prolonged viral load does not necessarily mean infectiousness, at least not in healthy, otherwise immunocompetent patients [9]. Most patients with mild and moderate forms of COVID-19 are no longer infectious at 10 days after symptoms onset. They can, however, test positive for SARS-CoV-2 by PCR via detection of non-viable RNA in nasopharyngeal specimens, up to three months (or longer) after illness onset [8,9]. It is not the same situation in immunocompromised patients, where replication-competent virus can be found even at 60 days after symptom onset [10,11,12,13]. As to why we have prolonged viral load, there are several hypotheses. Whilst there are studies that associate viral loads, viral duration, and viremia with disease severity [14,15], others fail to demonstrate this connection [6]. Even more, one study found an inverse correlation between disease severity and duration of RT-PCR positivity [16].

Therefore, this article aims to evaluate the number of days required by patients with mild and moderate forms of COVID-19 (generally those who do not need hospitalization) to reach undetectable levels of SARS-CoV-2 RNA in the upper respiratory tract specimens (nasopharyngeal swab). As a secondary outcome, we sought to determine a correlation between different patients characteristics (age, comorbidities, etc.) associated with a longer positive RT-PCR test, as this could result in longer period of contagion and infectivity.

## 2. Materials and Methods

Study design: It is a retrospective observational study conducted in Cluj-Napoca Rehabilitation and Recovery Hospital, between April and June 2020. During this period, the hospital was a COVID-19 support hospital, meaning that they had to keep the patients with mild and moderate forms of COVID-19 hospitalized until they had a negative RT-PCR test according to a ministerial order [17]. A mild COVID-19 patient was a patient with mild symptoms without pneumonia; a medium severity COVID-19 patient was a patient with symptoms and non-severe pneumonia (not requiring oxygen therapy). After a period of 14 days, tests were performed at a frequency of every 3 to 5 days. The reason for the short recruiting period is the fact that only during that time the RT-PCR testing was necessary at such a rate, and only in the beginning the negative testing was compulsory for discharge. Afterwards, the rules have changed. To prevent biases, we decided to take patients only from that period, hence the small sample size.

Study population: A total of 70 patients with mild and moderate, confirmed SARS CoV-2 infection were enrolled in the study. Inclusion criteria included: all patients over 18 years old, and a SARS-CoV-2 RT-PCR positive test from nasal swab. Exclusion criteria included: schizophrenia, severe psychological disorder, such as suicidal tendencies, with other infectious diseases, such as active tuberculosis, and patients who refused to participate in the study. The patients were kept in the hospital until they had two negatives RT-PCR tests at 24 h intervals. If the two consecutive negative tests were not obtained, the patient was not discharged. All patients signed an informed consent in the presence of one doctor and one nurse. The informed consent was afterwards sent via email to be kept in the patient’s chart and the original consent was later attached. The study was approved by the Ethics Committee of the “Clinical Hospital of Recovery Cluj-Napoca” number 28/27 April 2020.

Data collection: Demographic and epidemiological data (sex, age, comorbidities), blood count, chest X-ray interpretation, and the number of days from the first positive RT-PCR until two negative tests were collected for each patient from the electronic medical records. Chest X-ray interpretation was made by the two radiologists that work in the clinic; for interpretation accuracy, they both examined patients’ chest X-ray. Nasopharyngeal samples were collected by doctors in the morning using swabs and immediately placed in standard viral transport medium tubes and taken to the laboratory. The samples were collected from both nasal cavities of all patients. The analyzes were performed using ROTORGENE Q (real-time PCR). The kits used were GeneFinder COVID-19 PLUS RealAmp kit; manufacturer: ELITechGroup, detection limit: 10 copies/reaction. All tests were processed in the same laboratory, by the same team.

Statistical analysis: Current data was gathered using the Microsoft Excel 2020 program. Data was analyzed and interpreted using the IBM Microsoft SPSS v26.0 program. Normality of distribution was tested using the Kolmogorov–Smirnov test. Mean values were compared using the T-test for independent variables. Mean values are expressed with standard deviation, median values with quartiles. Correlation between quantitative variables were assessed using the Spearman Rho test for abnormally distributed data and Pearson test for normally distributed data. Median values were compared using the Mann–Whitney U test. Qualitative variables were expressed in numbers/frequencies and percentages. A subgroup analysis considering disease severity (mild or moderate) was made.

## 3. Results

A total of 70 patients with mild and moderate COVID-19 hospitalized in the Rehabilitation and Recovery Hospital from Cluj Napoca were enrolled in the study. Median age was 46 years old, with younger people in the mild COVID-19 group. Most patients were female. Mean hospitalization duration was around 26 days with no difference in terms of disease severity. Among patients with moderate COVID-19, more had chronic cardiac and liver diseases and a more important inflammatory syndrome during hospitalization (higher D-dimers, ferritin, and C-reactive protein values) (see Table 1). Despite higher values of inflammatory markers in the moderate COVID-19 group, there was no difference in viral persistence.

**Figure 1:**
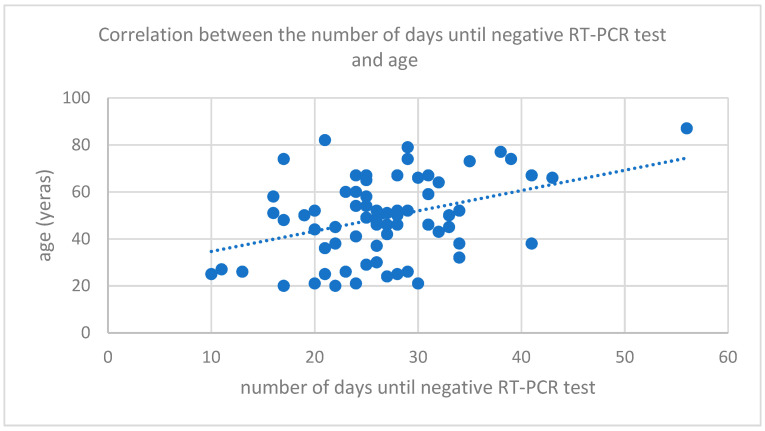
Correlation between number of days until negative RT-PCR test and age.

**Figure 2:**
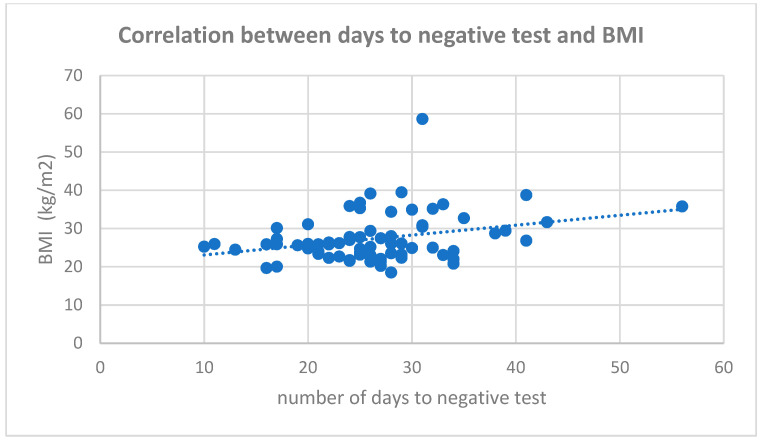
Correlation between days to negative test and BMI.

Factors associated with longer hospitalization, and therefore longer period of positive test, were age, body mass index, male gender, and chronic condition such as cardiac, renal disease, liver, and neurological disease (see Table 2). There was a mild to moderate correlation between age and body mass index (see Figure 1 and Figure 2).

## 4. Discussions

A total of 70 patients with mild and moderate forms of COVID-19 were enrolled in our study. Number of days with traceable viral load was 25.93 (±6.02) days in patients with mild COVID-19 and 26.97 (±8.30) in moderate form (*p* = 0.72). Characteristics associated with prolonged positive RT-PCR test from the nasal swab (therefore prolonged viral load) in our study were old age, high BMI values, male gender, and cardiac, liver, and renal chronic disease, i.e., mostly risk factors for severe COVID-19 disease. Persistence of viral RNA for a longer period is associated, according to published data, either with a high viral load and delayed viral clearance (in patients with severe disease) [18,19] or inappropriate immune response unable to promote RNA virus clearance [18]. Prolonged viral RNA detection, however, does not indicate prolonged infectiousness. According to the available data, the infected individuals are more contagious in the initial stages of illness when SARS-CoV-2 RNA levels in the upper respiratory specimens are the highest. After 7 up to 10 days of illness, disease transmission is highly unlikely, especially for immunocompetent patients without severe infection [5,19,20,21,22,23,24,25]. A study from China estimated that infectiousness peaked two days before symptoms onset and one day after and declined within seven days [21]. Another study that evaluated over 2500 close contacts of 100 patients with COVID-19 in Taiwan reported that there were no infections documented in the 850 contacts whose exposure was after 6 days of symptoms onset [25]. Even more so, analyzing over 280 cases from a hospital in northern Italy, the authors [26] observed over time a reduction of patients with COVID-19 requiring intensive care, along with decreasing median values of viral load. They noted an increasing in cycle threshold from a median value of 24 (IQR 19–29) to 34 (IQR 29–37) between March and May, with a statistically significant difference between March and April. Their conclusion was that low-transmission settings expose people to a lower viral load, therefore a less severe form of disease. Severe form of disease therefore leads to a high and prolonged viral load [26]. The duration of viral replication is however uncertain. Although most individuals with COVID-19 are no longer infectious after 10 days of symptom onset (for mild and moderate forms) and 20 days for those with severe or critical illness from COVID-19, recovered persons can still have a positive RT-PCR SARS-CoV-2. This can happen due to detection of non-viable RNA in nasopharyngeal specimens for up to three months after illness onset or even longer. Severely immunocompromised patients may produce replication-competent virus even after 20 days after symptoms onset and may require, as the CDC recommends, “additional testing and consultation with infectious diseases specialists and infection control experts” [27]. Differentiating between prolonged viral shedding of non-infectious RNA and persistent replicating viable virus can be difficult to determine without full evaluation of a patient’s clinical picture and timeline. To provide appropriate level of guidance for precautions and treatment, testing by PCR and analysis of cycle threshold values may provide key findings of viral replication, indicating the need for evaluation of additional treatment and maintaining isolation status in healthcare settings [17]. According to Carmo et al. [18], viral persistence for more than 2 weeks should not be associated with disease severity but rather with a weaker immune response from the host. On the other hand, severe disease has been associated with higher viral load, exaggerated response from the host, and prolonged viral detection in respiratory samples [4,5]. Nevertheless, there are data that show a longer RNA viral load in asymptomatic and patients with mild form of disease [4,6,7]. As virus viability has not been tested in the above-mentioned studies, we cannot say if it was replicable or not. It is worth mentioning that low IgG titers were found in the analyzed patients. This might be an argument as to why viral RNA clearance needs a longer period in these patients [5,19]. Both cellular and immune response might have a role in determining the speed of viral clearance as low white blood cells (leucocytes, eosinophils, and lymphocyte) count have been associated with a worse prognosis. According to Chang et al. [28] however, low leucocyte count has been associated with mild forms of COVID-19 and complete recovery. In our study, there was no statistically significant correlation between inflammatory status, evaluated though typical COVID-19 markers (D-dimers, CRP, ferritin etc.) at hospitalization and days of viral loads. Inflammatory markers were, however, higher in the moderate COVID-19 group. Age, male gender, and obesity, along with several chronic conditions, were associated with prolonged viral load. More severe forms of COVID-19 have been described in the male gender [28]. This gender dependent disease severity after infection was also reported during the SARS and MERS pandemic and seems to be related to differences in hormone levels [29,30,31,32]. The duration of virus persistence seems to be significantly longer in men than in women as also shown in our paper. Chronic respiratory conditions such as COPD and asthma were not among the conditions associated with prolonged viral persistence load. This might be due to their inhaled corticosteroids treatment [31] or to the fact that considering their chronic respiratory conditions, they were more cautious than usual [9,32,33]. Disease severity had no impact on viral clearance in this study. Longer viral load was observed among elderly patients, as their immune response might be diminished. Cardiac, neurological, and renal chronic disease were also associated with longer duration of viral load. One might argue that in this case we cannot eliminate age as a cofounder, as these conditions are very common among elder patients. It must be highlighted that our patients are relatively young as median age in the moderate COVID-19 group is 57 years old. This might then raise the problem that maybe they have had previously impaired health, but this will only confirm the hypothesis of impaired immune response in long RNA SARS-CoV-2 carriers. Limitations of the study are the retrospective nature, small sample size, and the fact that the virus replication cycle thresholds were not determined, so we cannot say if it was a replicating virus or not. Another limitation could be the fact that we did not determine the circulation variant, as this could also influence disease severity. Considering the time frame, it is the beta variant, but test have not been performed. It does, however, reinforce some facts, i.e., obesity, age, and male gender particularly are associated with longer persistence of viral load. As we do perform a lot of RT-PCR SARS COV-2 testing and among individuals tested positive some have passed through the disease completely asymptomatic, it would be interesting to see how many of them are still infectious (have replication virus) and/or need treatment. According to existing data, it seems that only severely immunosuppressed patients (neoplastic disease, patients receiving chemotherapy) can be infectious after a very long period, but considering the length of viral load in patients with chronic liver and renal disease, why cannot they be considered candidates?

## 5. Conclusions

In mild and moderate forms of COVID-19, there is no difference in the duration of viral load persistence. Factor associated with longer viral shedding in these patients are older age, obesity, males, and patients with cardiac, liver, renal, and neurological chronic conditions. Although according to the existing data they should not be considered infectious, we cannot exclude the possibility of viral replication, especially in patients with chronic conditions as apparently host immune response has an important role in viral clearance. Further research is required.

## Figures and Tables

**Table 1:** Patients’ characteristics considering COVID-19 form.

Parameters		Mild (*n* = 27)	Moderate (*n* = 43)	*p* Value
Age		35.78 (±14.23)	57.23 (±13.89)	0.001
Gender	Male	10	21	0.33
	Female	17	22	
Symptoms	Absent	9	20	0.03
	Present	3	38	
BMI		25.49 (±3.47)	27.74 (±7.30)	0.13
No days with traceable viral load		25.93 (±6.02)	26.97 (±8.30)	0.72
Smoking	Never	26	37	0.25
	Former	1	2	
	Current	0	4	
Chronic cardiac disease	Yes	1	23	0.001
	No	26	20	
Chronic pulmonary disease	Yes	0	3	0.06
	No	27	40	
Neurological diseases	Yes	1	2	0.62
	No	26	41	
Chronic renal diseases	Yes	2	6	0.47
	No	25	37	
Chronic hepatic diseases	Yes	0	5	0.04
	No	27	38	
Neoplastic diseases	Yes	1	5	0.11
	No	26	38	
Eosinophils		0.05 (0.005, 0.12)	0.04 (0.004, 0.09)	0.57
Lymphocyte		1.85 (1.4, 2.42)	1.52 (1, 1.89)	0.05
Ferritin		122.95 (21.3, 513.5)	323 (140, 707)	0.02
D-dimers		0.39 (0.28, 0.63)	0.76 (0.53, 7.71)	0.01
C reactive protein		0.48 (0.19, 2.08)	2.72 (0.83, 9.25)	0.002

**Table 2:** Clinical characteristics associated with longer RT-PCR SARS-CoV-2 positive testing.

Parameters		Days Until Negative RT-PCR Mean ± SD	*p*-Value
Chronic cardiac disease	Present	28.67 ± 9.375	0.001
	Absent	25.39 ± 6.143	
Chronic pulmonary disease	present	22.33 ± 6.110	0.33
	absent	26.7 ± 7.699	
Neurological diseases	present	37.33 ± 16.197	0.01
	absent	26.03 ± 6.908	
Chronic renal diseases	Present	35.20 ± 14.096	0.004
	absent	25.85 ± 6.662	
Chronic liver diseases	present	31.50 ± 11.402	0.04
	absent	25.87 ± 6.903	
Neoplastic diseases	present	28.83 ± 6.706	0.44
	Absent	26.30 ± 7.747	
Diabetes	present	26.46 ± 5.897	0.97
	absent	26.53 ± 8.045	
Gender	Number		
Female Male	39	28.15 ± 7.375	0.04
	31	24.45 ± 7.611	

## Data Availability

The data presented in this study are available in the manuscript. Additional raw data are available on request from the corresponding author.
